# Quality of Life of Pediatric Patients with Chronic Intestinal, Liver, and Pancreatic Diseases During the COVID-19 Pandemic

**DOI:** 10.3390/healthcare12232405

**Published:** 2024-11-29

**Authors:** Irina Dijmărescu, Andreea Maria Iordache, Daniela Păcurar, Elena Roxana Matran, Alexandra Coroleucă, Cristina Adriana Becheanu

**Affiliations:** 1 Department of Pediatrics, Faculty of Medicine, Carol Davila University of Medicine and Pharmacy, 020021 Bucharest, Romania; 2 Grigore Alexandrescu Emergency Children’s Hospital, 011743 Bucharest, Romania

**Keywords:** chronic digestive diseases, life quality, COVID-19, pandemic, children, Romania

## Abstract

Background/Objectives: Children with chronic diseases and their families face significant challenges to their quality of life compared to the rest of the population, and the COVID-19 pandemic has been a greater challenge for them. Our research aimed to identify key factors affecting their quality of life. Methods: We conducted a cross-sectional study in the Department of Pediatrics of the “Grigore Alexandrescu” Emergency Children’s Hospital in Bucharest on a group of 47 pediatric patients aged 9 years or older, diagnosed with chronic liver, gastrointestinal, and pancreatic diseases. Results: Children reported that their quality of life was not significantly affected by the pandemic due to the inability to see their family members and friends (89.36%), online education (67.5%), and social distancing (50%). Results showed that parents’ perceptions of the COVID-19 pandemic had a significant negative correlation with their quality of life (r = −0.35, *p* < 0.01); also, parents’ perceptions of the quality of life had a high negative significant correlation with their children’s mental well-being (r = −0.67, *p* < 0.001). Interestingly, children’s mental well-being did not have a significant correlation with their perceptions of quality of life (r = −0.02, *p* > 0.05). In the context of the COVID-19 pandemic, parents were concerned about their family well-being. Parents with higher scores for the perception of COVID-19’s impact tended to have lower scores for health-related quality of life. Also, the parents’ quality of life accounted for 44% of the variance in their children’s mental well-being. Conclusions: Even if the medical issue of the COVID-19 pandemic was not a concern for either children or their parents, many families were anxious about the perceived lack of dependability of the medical system. Addressability was delayed in many cases because of this, and regular medical care is essential for patients with chronic diseases.

## 1. Introduction

The World Health Organization defines health as a “complete state of physical, mental, and social well-being, not merely the absence of disease or infirmity” [1]. It is highlighted, therefore, that the evaluation of health and the quality of medical acts also implies the assessment of general well-being, which can be fulfilled by applying subjective questionnaires to determine the quality of life in general (QoL-Quality of Life) and health-related quality of life (HRQOL). These parameters are extremely useful in medical practice as their improvements or increases identify quality healthcare, which contributes to adequate diagnosis and treatment.

Pediatric patients (especially those with chronic diseases) are more sensitive regarding the quality of life: symptoms per se, relapses, multiple treatments, and their side effects, but also changes specific to pediatric age (growth failure and pubertal development, dietary restrictions and required surgical interventions in the context of disease progression, and the impact on school activities and social life), may negatively influence their quality of life. Moreover, their suffering majorly impacts their families and especially their parents. A chronically ill child can be a major source of stress for families, both socioeconomically and emotionally [2].

A 2021 study that assessed the financial burden in families of children living with life-limiting conditions highlighted the fact that not only direct costs (generated by investigations and treatment) are contributing, but so are indirect ones (transportation, accommodation, nutrition, psychotherapy, work absenteeism, changes in productivity at work, special education, etc.) [3].

Although, at the time we conducted this research, no studies on large groups to evaluate the impact of the pandemic on the pediatric population were available, the factors identified in the literature as having the greatest impact on children were the inability to see their family members and friends, online education, and social distancing [4,5].

To date, there have been numerous published studies focusing on the quality of life of children and adolescents during the pandemic in different geographical areas. A study conducted in Germany in 2022 including healthy children and adolescents revealed that two-thirds of them were highly burdened by the pandemic and that they experienced a significantly lower health-related quality of life and more mental health issues [6]. Similar results were reported in a study from Italy from 2022 [7]. Studies including adult subjects with inflammatory bowel disease had contradictory results regarding their quality of life. In Saudi Arabia, the pandemic seems not to have influenced the quality of life at all, and similar results were drawn when analyzing a population in Holland during the first lockdown [8,9]. In Romania, a study analyzing adult patients with inflammatory bowel disease reported a significant decrease in their quality of life during the pandemic compared to the period before [10].

Given the contradictory results and the fact that the presented studies were carried out in the adult population, we cannot hypothesize that the same conclusions can be drawn for children.

The COVID-19 pandemic and fears of SARS-CoV-2 infection have been a bigger challenge for children with chronic diseases and their families when compared to the rest of the population—difficult access to medical care, telemedicine, questions regarding the repercussions of SARS-CoV-2 infection on the underlying disease, and, also, social distancing and online schooling have had a significant impact on patients’ and families’ quality of life [11,12,13].

This article presents research that aimed to evaluate the quality of life of pediatric patients with chronic gastrointestinal diseases and their families during the first year of the COVID-19 pandemic. By identifying factors with greater impacts on the quality of life, medical professionals can understand the difficulties their patients face and subsequently develop and implement stress managing strategies for crisis situations, aiming at improving medical care. Even though things have considerably improved following the COVID-19 pandemic regarding social distancing, there are still important lessons to be learned from that experience, considering that pediatric patients with chronic gastrointestinal diseases are often more susceptible to infectious diseases.

As future directions, comparative studies focusing on the quality of life of pediatric patients with chronic gastrointestinal diseases during and after the COVID-19 pandemic may be conducted, analyzing aspects that could be improved following this experience.

## 2. Materials and Methods

We conducted a cross-sectional study in the Pediatrics Department of “Grigore Alexandrescu” Emergency Children’s Hospital in Bucharest, Romania. In the Department of Pediatrics, we evaluated patients with gastroenetrological, liver, and pancreatic diseases. The study group included 47 pediatric patients, aged 9 years and older, previously diagnosed with chronic liver and gastrointestinal diseases: inflammatory bowel disease (IBD)—ulcerative colitis and Chron’s disease, autoimmune liver disease, Wilson’s disease, and pancreatitis. The sample in our study was a convenience one. All patients evaluated and treated in our department for inflammatory bowel disease, chronic liver disease (autoimmune and Wilson’s disease), and chronic pancreatitis were included in the research, and since the medical part was not evaluated, all patients were eligible. Considering that they were chronic patients, with whom we had had a long collaboration, all the patients responded to the request to fill out the questionnaire. Some patients were in elementary school and others in high school. We considered age 9 appropriate for the child to understand and be able to answer the questions themselves without the help of their parents. Children learn how to read and write in Romania starting at age of 7.

Data were collected online by providing a link to a questionnaire created in Google Forms. In the beginning, the questionnaire included informed consent in which the purpose of the research was described. Also, a randomly allocated alphanumerical code was attributed to each family and was introduced by both the child and the primary caregiver (one of the parents—we will continue to refer to them as ‘parent’). Participants were then asked to provide basic demographic information (child’s age and gender). Participation was anonymous and completing the questionnaire took an average of 5 min for the child and 10 min for the parent.

The questionnaire was structured in two parts: the first part was addressed to the child and the second part to the parent. Children were administered two questionnaires: “Life quality during COVID-19” and “Warwick-Edinburgh Mental Well-being Scale (WEMWBS)”. The parents had to fill in several questionnaires: “Perception of COVID-19”, “Pediatric Quality of Life Inventory™ (PedsQL™)”, “Parents’ perception of the child scale”, and “The family’s perception of the child’s well-being”. All measures were administered in Romanian language.

Psychometric instruments are defined by two characteristics—reliability (proper measurement) and validity (the instrument can be used for measuring certain data). Cronbach alpha (based on interitem correlation) was measured to evaluate reliability. The accepted 0.7 threshold was exceeded for all instruments. To test the validity of the results, Confirmatory Factor Analysis (CFA) [14] was used.

The perception of COVID-19 pandemic was measured using a unidimensional scale that consists of 6 items (e.g., “What impact do you expect the COVID-19 pandemic to have on your children’s education?”, Cronbach’s alpha = 0.76). All items were scored on a Likert scale from 1 (not worried) to 5 (extremely worried). Higher scores showed higher levels of concern regarding COVID-19 pandemic.

Health-related quality of life was assessed using Pediatric Quality of Life Inventory™ (PedsQL™) [15]. The scale has six dimensions for parent functioning and two dimensions for family functioning. The dimensions for parent functioning evaluate physical functioning (6 items, e.g., “I feel tired during the day”, Cronbach’s alpha = 0.90), emotional functioning (5 items, e.g., “I feel sad”, Cronbach’s alpha = 0.91), social functioning (4 items, e.g., “I feel isolated from others”, Cronbach’s alpha = 0.83), cognitive functioning (5 items, e.g., “It is hard for me to concentrate when doing an activity”, Cronbach’s alpha = 0.90), communication (3 items, e.g., “I feel like others don’t understand my family’s situation”, Cronbach’s alpha = 0.70) and worry (5 items, e.g., “I am worried because I don’t know if the treatment will work for my child’s condition”, Cronbach’s alpha = 0.79). The dimensions for family functioning evaluate daily activities (3 items, e.g., “Daily family activities require more time and effort”, Cronbach’s alpha = 0.77) and family relationships (3 items, e.g., “I found that there is a lack of communication between family members”, Cronbach’s alpha = 0.91). All items were scored on a 5-point rating scale ranging from 0 (never a problem) to 4 (always a problem). Lower scores indicate lower negative impact and better functioning.

Parents’ perceptions of children in the context of the COVID-19 pandemic were measured using a 7-item unidimensional scale (e.g., “How do you perceive the child’s level of satisfaction with school in the current epidemiological conditions?”, Cronbach’s alpha = 0.80). All items were scored on a 5-point rating scale ranging from 1 (very dissatisfied) to 5 (very satisfied). Higher scores indicate higher levels of satisfaction.

The family’s perception of the child’s well-being was assessed using a unidimensional scale with 6 items (e.g., “She/he feels happy”, Cronbach’s alpha = 0.88). All items were scored on a Likert scale from 1 (never) to 5 (always). Higher scores showed higher levels of well-being.

Life quality perception during COVID-19 was assessed using a unidimensional scale with 7 items (e.g., “On a scale from 0 to 10, how much were you affected by the need imposed by the pandemic to wash your hands frequently?”, Cronbach’s alpha = 0.83). All items were scored on a Likert scale from 0 (not at all) to 10 (very distressed). Higher scores showed higher levels of distress.

Mental well-being was measured using Warwick–Edinburgh Mental Well-being Scale [16]. The scale comprises 14 items (e.g., “I’ve been feeling optimistic about the future”, Cronbach’s alpha = 0.90) and it is unidimensional. All items were scored on a 5-point rating scale ranging from 1 (never) to 5 (always). Higher scores indicated higher levels of mental well-being.

We need to note that some questionnaires were used in their original validated forms, which specified a 5-point Likert scale, while others required greater granularity and utilized a 10-point scale to capture more detailed responses. We kept the different Likert scales in order to meet the specific requirements of the study.

Given that validated scales to measure the COVID-19 perception were not available, as the pandemic was ongoing during the time this research was performed, the authors created their own scales to evaluate COVID-19-related constructs: “Life quality during COVID-19”, “Perception of COVID-19”, “Parents’ perception of the child scale”, and “The family’s perception of the child’s well-being”. The authors used both deductive and inductive methods in developing the items for the above-mentioned scales. These scales provided an initial tool for measuring COVID-19 perception. Also, our results show promising psychometric properties for internal consistency and factorial structure.

All statistical analyses were performed in R Studio (2023.12.0+369 “Ocean Storm” (33206f75, 2023-12-17)) [17] using psych [18], lavaan [19], and ggplot2 [20]. To analyze the validity of our measures, we used Confirmatory Factor Analysis (CFA) [14] for all the questionnaires we administered. Four indices were used to assess goodness of fit: Comparative Fit Index (CFI), Tucker–Lewis Index (TLI), Root Mean Square Error of Approximation (RMSEA), and Standardized Root Mean Square Residual (SRMR). An acceptable fit was considered for values above 0.90 for CFI and TLI [21] while a very good fit was considered for values above 0.95. Also, values of 0.08 or lower for RMSEA and SRMR were considered acceptable [22].

Considering the relatively small sample and the number of the free parameters resulting from our measures, which could not lead to a reasonable N:q ratio (in our case, 47 participants), we evaluated the measurement model for PedsQL scale (which consists of 34 items) using parcels. As Marsh and Hocevar (1988) pointed out, the participant: item ratio should be explicitly considered as lower ratios could result in instability for the factor solution [23]. Therefore, the rationale for using parcels was to improve the accuracy and stability of the parameter estimates [24,25]. The parcels were created as follows: for parent functioning, the items from each subscale were summed together, resulting in 6 parcels— physical functioning, emotional functioning, social functioning, cognitive functioning, communication, and worry—and for the family functioning, resulted in 2 parcels: daily activities and family relationships.

## 3. Results

**Figure 1:**
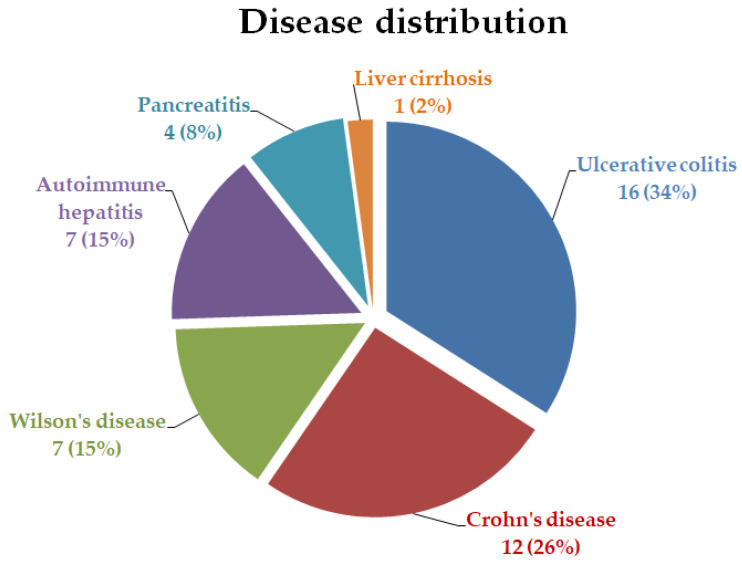
Disease distribution.

We included, in the study group, 47 pediatric patients aged above 9 years old, previously diagnosed with chronic liver and gastrointestinal diseases. The mean age of the patients was 14.7 years and the genders were equally represented (M/F = 1/1). Regarding the chronic disease, 60% were diagnosed with IBD (34% ulcerative colitis and 26% Crohn’s disease), 15% had autoimmune liver disease, 15% Wilson’s disease, 8% chronic pancreatitis, and 2% liver cirrhosis. (Figure 1).

The consequences of the COVID-19 pandemic (the social distancing, online education, and inability to see family members out of the household as frequently) had a negative impact on the perceived well-being for 80.85% of the children included in the study group. The inability to see their friends had a negative impact on their perceived well-being for 89.36% of them.

Also, 90% of the parents reported to be worried about the family well-being in the context of the COVID-19 pandemic. When looking at the changes imposed in our society, the parents reported, as negative factors, the following: 50% of them highlighted social distancing, 25% of them isolation, 67.5% of them online education, and 30% of them a reduced family income secondary to the pandemic, and 27.5% were dissatisfied because of the postponed holidays. Eighty percent of the parents were worried about reaching inpatient medical care.

Table 1 includes the CFA analyses for the measures used in our study: Life Quality COVID-19, Mental Well-Being, COVID-19’s perception, PedsQL, Parents Perception of their children and General Family Perception of their children. For establishing the model’s goodness of fit, cut-off criteria provided by Hu and Bentler (1999) were used [22].

The fit indices for the measurement model showed a good and acceptable fit with the data, except the RMSEA, which tended to be above the acceptable threshold of 0.80. As Hu and Bentler (1999) stated, the RMSEA over-rejects models at small sample sizes. Despite this, the other fit indices consistently indicated a good fit. To further validate the model, we also calculated the Chi Square to Degrees of Freedom (CMIN/df) ratio, and for all our measures, the value was below 2 as the literature recommends [22]. Given the results of CFA and reliability, the measure had adequate psychometric properties.

The means, standard deviation, internal consistency (Cronbach’s alpha), and inter-correlations among the measured variables were calculated. The results are reported in Table 2.

The correlation analysis showed that there was a significant negative correlation between parents’ perceptions of the COVID-19 pandemic and their quality of life (r = −0.35, *p* < 0.01). When analysing the impact of the mental well-being of children on their perception of quality of life, it was not statistically significant (r = −0.02, *p* > 0.05). At the same time, parents’ perceptions of the quality of life had a high negative significant correlation with their children’s mental well-being (r = −0.67, *p* < 0.001).

Linear regression was used to test the relation between children’s life quality perception during COVID-19 and their mental well-being. The results showed a non-significant relation with R^2^ = 0.05; F (1, 45) = 0.02 (*p* > 0.05).

Further on, we tested the relation between parents’ perceptions of COVID-19 and health-related quality of life. The results showed that 16.1% of the variance in the health-related quality of life is explained by the perception of COVID-19. Thus, the fitted regression model that resulted was as follows:

Health-related quality of life = 110.88 − 1.91 ∗ COVID-19’s perception(1)

**Figure 2:**
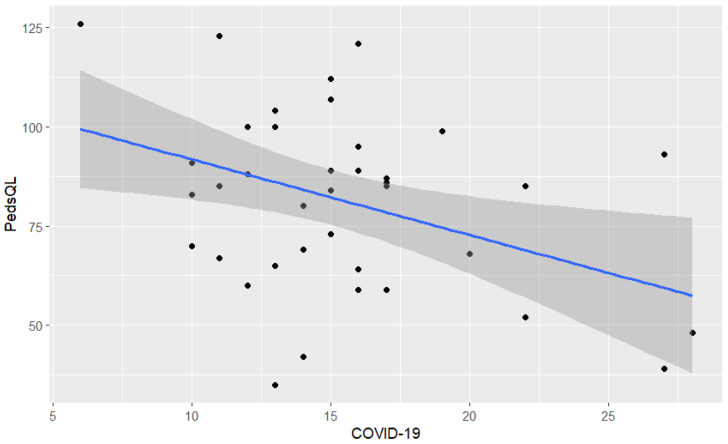
The relation between perception of COVID-19 and parents’ health-related quality of life (PedsQL).

The data supported our model, which was statistically significant with R^2^ = 0.16 and F (1, 38) = 7.28 (*p* < 0.05) (Figure 2). 

The results showed that the parents with higher scores for perception of COVID-19’s impact tended to have lower scores for health-related quality of life.

Next, linear regression was used to evaluate whether parents’ quality of life (measured using PedsQL) significantly predicted their children’s mental well-being.

The fitted regression model that resulted was as follows:

Children’s mental well-being = 78.01 − 0.27 ∗ parents’ quality of life(2)

**Figure 3:**
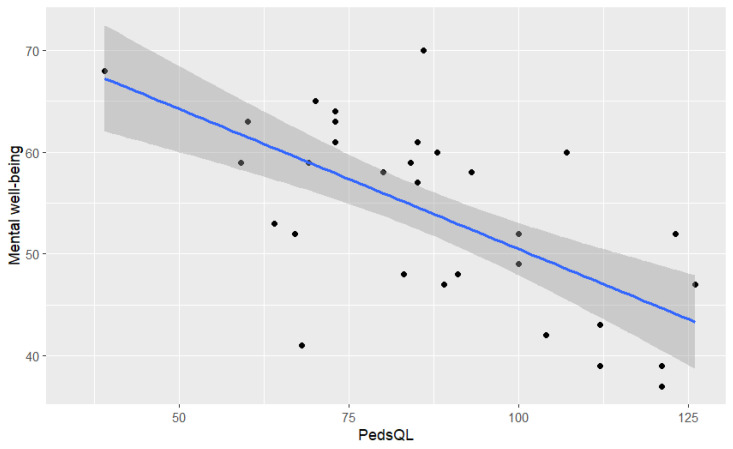
The relation between parents’ quality of life (PedsQL) and children’s mental well-being.

The parents’ quality of life accounted for 44% of the variance in their children’s mental well-being (R^2^ = 0.44, F (1, 38) = 30.19, *p* < 0.000). The relation is presented in Figure 3.

## 4. Discussion

There are few publications in the medical literature that have evaluated the impact of the COVID-19 pandemic on the mental health and well-being of pediatric patients with chronic gastrointestinal diseases.

Our experience in caring for children with chronic gastrointestinal diseases (IBD, chronic hepatitis, chronic pancreatitis, celiac disease, etc.) has shown that the quality of life in these cases is majorly influenced by concern regarding the disease and symptoms that impact well-being and limit access to age-appropriate social activities. The need for regular medical check-ups (moreover when implying blood tests and other painful invasive evaluations), chronic treatment and the fear of adverse reactions or surgical interventions that may be needed during the course of the disease, growth impairment, and sexual development also contribute to the issues. Concern regarding uncertainty about the future is added, along with limited choices for occupation, school absenteeism during flares, and obstacles in socializing and spending time with friends (camps, trips, going out to restaurants, etc.).

This aspect has become more apparent during the COVID-19 pandemic, when we saw that most patients with chronic gastrointestinal diseases had had severe relapses. To assess the impact of the quality of life on triggering relapses, we evaluated the quality of life of children and their parents using the PedsQL Family Impact Module.

One of the aspects that has been reported by more than 80% of patients as being of negative influence when speaking of the quality of life is the social distancing imposed by the pandemic. Social distancing included several aspects—physical distancing (more than 2 m among people), 14-day quarantine for suspects, and a general quarantine during the public-health state of emergency. An interesting observation is that limiting contact with friends has a greater impact on them than limiting contact with extended family (89.36% vs. 80.85%). For our patients, this meant a severe limitation of spending time in school and going out with friends, being sentenced to solitude at home. Research published in May 2022 by Beth et al. concluded that “Physical distancing and isolation, which for children with chronic illnesses may persist longer, may exacerbate mental health concerns, and access to behavioral healthcare is essential” [26].

American researchers report that patients with chronic diseases account for about 20% of the pediatric population, and the pandemic and related actions to prevent the spread of the SARS-CoV-2 virus “increased their stress and reported their stress was related to isolation, lack of resources, and concern for the mental health of other children in the household” for nearly all patients [27]. But a study conducted by Courtwright et al. on 154 adolescent patients with chronic conditions showed that the psycho-emotional impact of the COVD-19 pandemic was not very important, except for exacerbations of the diseases, as long as proactive medical involvement was present (mostly nurses) [28].

Several publications in the medical literature show that patients with IBD have a lower HRQOL, lower social functioning, and higher rates of depression when compared to their peers [29,30,31].

Research conducted in the Netherlands and published in 2017 showed that young patients (10–20 years) with IBD had a lower HRQOL score when compared to healthy responders, especially because of school physical activity limitation. The severe course of the disease was associated with high parental stress and indirectly with lower HRQOL values in children with IBD [32].

In our country, research conducted at a Pediatrics Department from Cluj-Napoca demonstrated that IBD patients and their families are frequently undergoing psycho-social stress and that it should be detected early. The authors signal that by understanding the way the quality of life is impacted by these disturbances, the needs of IBD patients and their families will be better seen and in this way, intervention and support teams may be properly developed. A good patient–doctor relationship has a positive influence on the course of the disease when IBD is concerned, and addressing the psycho-social needs of these patients may increase compliance [33].

Research from Israel has shown that besides a great concern in respect to COVID-19 infection and worry regarding accessing healthcare, IBD patients evaluated reported an even greater social distancing and school absenteeism than the rules imposed by their government [34].

All these aspects influence the course of the disease. Severe progression is directly associated with the parents’ suffering and indirectly with a lower HRQOL in children and adolescents diagnosed with IBD. Parents’ suffering may be considered when managing pediatric IBD to raise the HRQOL in children [11].

There have been no pediatric studies regarding the quality of life in patients with chronic liver disease during the COVID-19 pandemic. A study carried out in the adult population with liver cirrhosis compared scores of the quality of life for patients before and during the COVID-19 pandemic, identifying higher scores during the pandemic: self-care was better during lockdown, their ability to conduct daily activities was improved, and the anxiety and depression were lower, but the pain and discomfort were at the same level [12]. The quality of life of pediatric patients with chronic liver disease might have also been higher during the pandemic, considering that parents were possibly able to better control their diets and manage medication, enhancing medical care. Also, closer monitoring may allow the early identification of signs of decompensated liver disease. In pediatric patients, because parents are able to be closer to their children, this aspect might be critical, unlike in the adult population.

During the pandemic, impositions such as the closure of schools nationwide had particularly strong impacts on families with children. In this regard, a previous study conducted on Romanian students revealed the fact that students encountered many difficulties during the process of online learning such as technical difficulties, poor interaction with teachers, and poor communication with their peers [4].

The impact of closing schools was also evaluated in our study, focusing especially on changes in family lifestyles and the impact on children and parents. More than two-thirds of our evaluated patients acknowledged that closing schools had negatively impacted their well-being.

A meta-analysis published in the United States evaluated the impact of closing schools during the pandemic and concluded that children and young people’s physical and social activities were severely affected. The reported effects were less exercise, less balanced diets, more screen time, and irregular sleep patterns [2].

A meta-analysis published in 2022 that analyzed 2830 studies focusing on the psycho-emotional impairment of healthy children and adolescents during the pandemic indicated severe problems regarding mental health in relation to reductions in school activity and the implementation of virtual education: higher suicidal risk, anxiety, depression, emotional disorders, stress, and sleep disorders [35].

Considering that in certain areas of Romania, access to technology and high-speed internet is difficult, many of the children, especially those coming from families with low socioeconomic levels, encountered difficulties regarding online school. However, for other children with very good technical skills and good access to technology, online school was an excellent way to maintain their academic performance and stay busy during such a stressful time.

What most impacted the life quality of the patients included in this research was the inability to socialize with their friends during the pandemic: more than 90% of the children questioned in our study reported that they had been negatively influenced by this aspect. The medical literature also highlights this aspect as being a stress factor for children and adolescents. Childhood friendships, especially in young children, are less stable and more likely to be disrupted only 50% of friendships are reported to be steady over one school year at age 5 while the percentage rises to 75% at age 10 [36].

Moreover, socializing among children often depends on gatherings planned by adults (for instance, play dates, meetings in the park, and leisure activities), and these opportunities were severely reduced during the pandemic. Maybe the most important factor is that friendships usually develop among children of the same age [37], suggesting that closing schools may have a direct impact on building and maintaining friendships.

A study published by Canadian researchers aimed to record children’s and adolescents’ perspectives regarding social life and friendships during the COVID-19 pandemic through qualitative interviews. A number of 67 children and adolescents were interviewed and the research concluded that most of all, they had missed their friends during the pandemic. Also, the authors concluded that online communication is convenient but does not replace face-to-face interactions. School represents an essential environment for socialization, and online schooling does not meet the same needs regarding daily social interactions [5].

If, for children, a lack of socializing has been the major stress factor, in our cohort, more than 80% of the parents of children with chronic diseases were significantly concerned about access to medical care—concern for relapse and the impossibility to reach the doctor and concern regarding the risk of infection. Other reported concerns were the long-time progression of the disease and how the children’s futures as adults would be affected (90% of the parents in our study reported to be worried for the family well-being), how schooling and school performance would be impacted (67.5% of the parents perceived online education as a negative factor), adverse reactions and the management of disease complications, the need for frequent and prolonged hospitalizations, the fear of discrimination and fear of higher economic needs required in the context of caring for a chronically ill child, the need for psychotherapy, and work absenteeism. Although, before conducting this research, we did not evaluate our patients’ quality of life in questionnaires, we are aware of these facts and their major impact on disease progression. Given the unknown responses of patients with chronic diseases to COVID-19 infection, and the unknown impact of immunosuppressive therapy (including biologic agents) on their response to the disease, parents were more careful in accessing healthcare, and in some cases resorted to auto medication, and they refused to attend follow-up visits [38].

In a Canadian study that evaluated 50 families with IBD children, 50% of parents reported concern regarding accessing healthcare because they were worried that they might be infected by SARS-CoV-2. However, because information was published on official sites and the rules were clearly transmitted by the authorities, they were encouraged to strictly follow the rules and address doctors with confidence when needed. School was the place considered most risky for children to contact SARS-CoV-2, and thus, social distancing and isolation were extended on their own initiative [34].

Research conducted in the USA that included 300 children with chronic diseases and their parents evaluated the stress level of parents. It was concluded that the pandemic had brought up, for parents, unheard-of levels of stress, particularly for the families of children with chronic diseases. The researchers reported high rates for clinical anxiety (44.6%) and depression (42.2%), higher than the rates reported in other studies. All this was cumulated with reduced access to healthcare. The authors highlighted the need for support of these patients, who are even more stressed than the parents of children without chronic diseases. Moreover, they emphasized that these problems would continue for the respective families for a long time after the end of the pandemic, so they would need further support [39].

In the post-pandemic period, we have noticed an increase in the number of cases of functional disorders such as functional abdominal pain in the pediatric population, as also reported by the campaign conducted by The European Society for Paediatric Gastroenterology, Hepatology and Nutrition (ESPGHAN) in collaboration with the General Paediatric Societies and the National Societies for Paediatric Gastroenterology, Hepatology and Nutrition (PGHAN) in Europe [40]. Children with chronic digestive diseases may also have associated functional disorders. Therefore, if children present with digestive symptoms that are not suggestive of a relapse of the underlying disease, we should consider the association of a functional disorder and refer them to psychological evaluation. Due to the collaboration with our psychologist colleagues, we have also managed to create support groups for children with chronic diseases. Considering that they are young patients, with long life expectancy, who will be exposed to other special contexts similar to the pandemic, we consider that all patients with chronic diseases must be psychologically evaluated and counseled periodically.

Also, the COVID-19 pandemic amplified demands on parents regarding balancing childcare and working from home. Research indicates that the pandemic resulted in an increased burden for mothers, but also that it created a novel situation in which parenting and working styles could be re-evaluated [41].

Although this may have led many parents to spend more time with their children, the measures also led to the disruption of established routines and enormous pressure for parents concerning educational activities. In the context of the pandemic, many parents reduced their work schedules, and therefore family income, and tried the difficult task of combining homework with childcare [42].

There is some evidence that suggests that parental stress surged at the beginning of the pandemic. Parents reported increased anxiety, depression, agitation, and sleep disturbances [2].

At the same time, isolation and the restriction of interpersonal contact are to a much lesser extent considered by parents to affect the quality of life (50%). On the other hand, parents can experience their own psycho-emotional problems, which they do not fully recognize or explain, but they can pass them on to their children, possibly causing certain parenting problems, excessive protective behavior, and the maintenance of post-traumatic stress problems in children [13].

Our results showed that parents’ perceptions of the COVID-19 pandemic negatively impacted their quality of life. Although, for children, the impact of this perception on their quality of life was not significant, parents’ perceptions of the quality of life negatively correlated with their children’s mental well-being. We hypothesize that when parents had a lower perception of the quality of life in relation with the COVID-19 pandemic, they did not perceive this as a threat, and subsequently, they did not focus on shielding their children from exposure to negative information, and this aspect might have contributed to children’s anxiety.

Also, the impact of the pandemic on the family, such as in the form of depression and anxiety in parents, was related to an increased risk of mental problems among children [43].

We consider that our research is important even if it included a small number of patients because it was the first study of this kind in our country, evaluating parental and pediatric patients’ stress in a population of children with chronic digestive diseases during the pandemic.

## 5. Conclusions

Social distancing and the lack of interaction with friends were perceived as major negative factors during the pandemic for children with chronic digestive diseases. At the same time, their parents were concerned about online education. Parents with higher scores for perceptions of COVID-19’s impact tended to have lower scores for health-related quality of life. Parents’ quality of life majorly impacted their children’s mental well-being. Although overall, the pandemic was a stressful period for both children and their families, we believe that they developed new coping mechanisms and increased their levels of resilience. In the case of new special situations in their lives, they will certainly be more prepared to find adapted solutions, of course, under the guidance of the medical staff and with the support of the psychologists with whom many have developed close relationships.

Even if the medical issue of the COVID-19 pandemic was not a concern for either children or their parents, many families were anxious about the perceived lack of dependability of the medical system. Addressability was delayed in many cases because of this, and regular medical care is essential for patients with chronic diseases.

## 6. Limitations

Despite the contributions of our study, several limitations should be considered. First, we used self-reporting questionnaires to measure our variables, which could have led to common method bias [44]. Second, we used a small sample size, which may have reduced the generalizability of the findings to a broader population. Additionally, statistical power is also limited in smaller samples.

## Figures and Tables

**Table 1:** Fit indices for measures.

Measure	Model	χ^2^	df	χ^2^/df	CFI	TLI	RMSEA (90% CI)	SRMR
Life Quality COVID-19	1 Factor *	21.25	13	1.63	0.94	0.91	0.12 (0.00–0.21)	0.08
Mental Well-Being	1 Factor **	110.07	72	1.53	0.89	0.87	0.11 (0.07–0.15)	0.09
COVID-19’s perception	1 Factor	11.28	9	1.25	0.96	0.93	0.09 (0.00–0.19)	0.07
PedsQL™	2 Factors	24.87	19	1.31	0.97	0.96	0.09 (0.00–0.17)	0.06
Parents Perception	1 Factor *	24.06	14	1.72	0.9	0.86	0.13 (0.03–0.22)	0.1
Family Perception	1 Factor	17.11	9	1.90	0.94	0.9	0.14 (0.02–0.24)	0.06

Note. * indicates 2 correlated errors; ** 4 correlated errors; CFI = comparative fit index; TLI = Tucker–Lewis index; RMSEA = Root Mean Square Error of Approximation; SRMR = Standardized Root Mean Squared Residuals.

**Table 2:** Descriptive statistics and correlations between the variables included in the study.

Variable Name	Mean	SD	1	2	3	4	5	6
1. COVID-19’s perception	14.98	4.47	(0.76)					
2. PedsQL	82.6	22.64	−0.35 **	(0.96)				
3. Parents Perception	18.92	3.69	0.1	−0.57 ***	(0.80)			
4. Family Perception	24.04	4.3	0.35 **	−0.78 ***	0.53 ***	(0.88)		
5. Life Quality COVID-19	46.71	16.37	−0.18	0.26	−0.22	−0.16	(0.83)	
6. Mental Well-Being	55.63	8.66	0.22	−0.67 ***	0.55 ***	0.78 ***	−0.02	(0.90)

Note. ** *p* < 0.01, *** *p* < 0.001; Values in parenthesis represent Cronbach’s alpha. The first two scales were administered to the children while the rest were administered to children’s parents.

## Data Availability

Data are contained within the article.
